# Supplementary material for: Women’s autonomy in healthcare decision-making and healthcare seeking behaviour for childhood illness in Ghana: Analysis of data from the 2014 Ghana Demographic and Health Survey
Source: PLoS One. 2020 Nov 9;15(11):e0241488. doi: 10.1371/journal.pone.0241488 (PMC7652316; doi:10.1371/journal.pone.0241488)
Supplement: Attachment — Submitted filename: Response to comments.docx [file pone.0241488.s002.docx]

Abdul-Aziz Seidu

University of Cape Coast, Ghana

Department of Population and Health

31/05/2020

Dear Editor,

Thank you for giving us the opportunity to revise our manuscript before sending it out for peer review. We have revised it based on the issues raised.

**1. Comment:** ......'women whose children weighed 50-100 kg, and 101-150kg were more likely to seek healthcare for childhood illnesses'

***Response: Please we have corrected this. We have corrected to “Child’s weight was recoded into below 2.5kg (1) and 2.5kg and above (2). This weight was generated based on studies that have considered a child’s weight less than 2.5kg as low birth weight and those 2.5kg and above as non-low birth weight [35-38].***

**2. Comment**: These weight ranges are not commensurate with childhood weight averages

***Response: Child’s weight was recoded into below 2.5kg (1) and 2.5kg and above (2). This weight was generated based on studies that have considered a child’s weight less than 2.5kg as low birth weight and those 2.5kg and above as non-low birth weight [35-38].***

**Comment**: 3. Please ensure that your manuscript meets PLOS ONE's style requirements, including those for file naming. The PLOS ONE style templates can be found at https://journals.plos.org/plosone/s/file?id=wjVg/PLOSOne_formatting_sample_main_body.pdf and <https://journals.plos.org/plosone/s/file?id=ba62/PLOSOne_formatting_sample_title_authors_affiliations.pdf>

***Response: Please we have formatted the manuscript to met PLOS ONE's style requirements***

**Comment**: 4. In your Methods section, please provide additional information about the  demographic details of your participants. In particular, please clarify how "child weight" was categorised, and ensure that the numbers reported are correct, as it seems unlikely that children aged 0-5 years weighted up to 200 kg.

**Response: *Child’s weight was recoded into below 2.5kg (1) and 2.5kg and above (2). This weight was generated based on studies that have considered a child’s weight less than 2.5kg as low birth weight and those 2.5kg and above as non-low birth weight [35-38].***

**Comment**: 5. Please correct your reference to "p=0.000" to "p<0.001" or as similarly appropriate, as p values cannot equal zero.

**Response: Please this has been corrected. Thank you.**

Yours Sincerely,

On behalf of the authors

Abdul-Aziz Seidu
